# Quantitative MRI using relaxometry in malignant gliomas detects contrast enhancement in peritumoral oedema

**DOI:** 10.1038/s41598-020-75105-6

**Published:** 2020-10-22

**Authors:** I. Blystad, J. B. M. Warntjes, Ö Smedby, P. Lundberg, E.-M. Larsson, A. Tisell

**Affiliations:** 1 Department of Radiology in Linköping and Department of Health, Medicine and Caring Sciences, Linköping University, Linköping, Sweden; 2 Centre for Medical Image Science and Visualization (CMIV), Linköping University, Linköping, Sweden; 3 Division of Cardiovascular Medicine, Department of Health, Medicine and Caring Sciences, Linköping University, Linköping, Sweden; 4 School of Technology and Health, KTH Royal Institute of Technology, Stockholm, Sweden; 5 Department of Radiation Physics and Department of Health, Medicine and Caring Sciences, Linköping University, Linköping, Sweden; 6 Department of Surgical Sciences, Radiology, Uppsala University, Uppsala, Sweden

**Keywords:** Cancer imaging, CNS cancer

## Abstract

Malignant gliomas are primary brain tumours with an infiltrative growth pattern, often with contrast enhancement on magnetic resonance imaging (MRI). However, it is well known that tumour infiltration extends beyond the visible contrast enhancement. The aim of this study was to investigate if there is contrast enhancement not detected visually in the peritumoral oedema of malignant gliomas by using relaxometry with synthetic MRI. 25 patients who had brain tumours with a radiological appearance of malignant glioma were prospectively included. A quantitative MR-sequence measuring longitudinal relaxation (R_1_), transverse relaxation (R_2_) and proton density (PD), was added to the standard MRI protocol before surgery. Five patients were excluded, and in 20 patients, synthetic MR images were created from the quantitative scans. Manual regions of interest (ROIs) outlined the visibly contrast-enhancing border of the tumours and the peritumoral area. Contrast enhancement was quantified by subtraction of native images from post GD-images, creating an R_1_-difference-map. The quantitative R_1_-difference-maps showed significant contrast enhancement in the peritumoral area (0.047) compared to normal appearing white matter (0.032), p = 0.048. Relaxometry detects contrast enhancement in the peritumoral area of malignant gliomas. This could represent infiltrative tumour growth.

## Introduction

Malignant gliomas are primary brain tumours with an incidence of approximately 5/100,000/year and in many cases a poor prognosis, with a median survival time of 12–15 months for grade IV glioblastomas^1,2^. MRI is an important tool in the diagnostic work-up as well as for treatment monitoring and during follow-up evaluation. Conventional MRI relies on visual assessment and evaluation by the radiologist, with contrast enhancement and peritumoral oedema being important features to describe. However, assessment of malignant gliomas is complicated due to their heterogeneous and infiltrative nature^3^, and in the post-treatment period, chemo- and radiotherapy can cause treatment related changes with a tumour-like appearance, which also complicates the MRI evaluation^4^. During recent years, there has been a rapid development towards application of more quantitative MRI techniques for brain tumour evaluation^5,6^, as well as efforts to find image correlates to physiological and molecular processes of brain tumours^7–10^ and to patient outcome^11–14^.

Due to their infiltrative nature, malignant gliomas are difficult to treat and to assess^3^. Surgery aims for maximum safe resection of the tumour, and complete removal of the contrast-enhancing portion is regarded as radical resection^15,16^. However, glioma infiltration extends beyond the visibly contrast-enhancing border of the tumour, and these changes are not easily differentiated from the peritumoral oedema on conventional MR images^17,18^. Using quantitative MR techniques, tumour infiltration can be analysed for diagnosis and prognosis^19–22^, and during recent years such new quantitative MRI sequences using relaxometry have been applied for brain tumour analysis in research^23–25^.

This study was performed to investigate the contrast-enhancing properties of malignant gliomas and their peritumoral oedema, using relaxometry to quantify contrast enhancement.

## Materials and methods

### Subjects

Twenty-five patients with radiological findings suggestive of high-grade malignant gliomas were prospectively included in the study from 2013 to 2016. This cohort has previously been reported in^25^. Patients were examined with MRI before surgery. The diagnosis of malignant glioma was confirmed with histopathological analysis after surgery. Three patients were excluded due to other diagnoses; one abscess, one lymphoma and one primitive neuroectodermal tumour (PNET). Tumour classification was made according to WHO 2007, which was clinical standard at the time of inclusions. Patient demographics are summarized in Table 1. Two patients were excluded from the analysis due to motion leading to invalid data sets. The local institutional review board of the Swedish Ethical Review Authority in Linköping approved the study (Dnr 2011/406-31), and informed written consent was obtained from all patients.

**Table 1:** Patient demographics and volume of manually delineated contrast-enhancing part of tumour including necrotic core in T1 3D-FSPGR GD.

Patient	Sex	Age	Diagnosis WHO 2007	Volume (mL)
1	M	63	Anaplastic oligodendroglioma III	84
2	M	71	Glioblastoma	11
3	F	58	Glioblastoma	76
4	M	73	Glioblastoma	17
5	F	57	Glioblastoma	6
6	M	65	Glioblastoma	113
7	F	61	Glioblastoma	4
8	F	65	Anaplastic oligodendroglioma III	24
9	M	69	Glioblastoma	21
10	M	34	Anaplastic oligodendroglioma III	1
11	M	50	Anaplastic oligodendroglioma III	5
12	M	79	Glioblastoma	100
13	M	68	Glioblastoma	84
14	M	43	Oligodendroglioma II	0.2
15	M	65	Gliosarcoma	26
16	M	46	Glioblastoma	26
17	M	72	Glioblastoma	92
18	M	76	Glioblastoma	31
19	F	45	Glioblastoma	19
20	M	54	Glioblastoma	49

Patient age, sex, the histopathological diagnosis according to WHO 2007 classification for brain tumours, and the volume of contrast enhancing tumour including the necrotic core in the 3D-FSPGR GD images.

### MRI protocol

Magnetic resonance data was acquired on a 3 T MR scanner (MR750, GE Medical Systems, Milwaukee, Wisconsin, US) using a 32-channel phased array head coil. Patients were examined according to the clinical protocol for brain tumour investigation, with the addition of the qMRI sequence SyMRI MAGiC (GE Healthcare) both before and after contrast agent injection. The clinical protocol for brain tumour investigation consisted of conventional axial T2WI-FLAIR, T1WI, T2WI, diffusion weighted images (DWI), dynamic susceptibility contrast (DSC) perfusion, T1WI-GD, and 3D-FSPGR (fast spoiled gradient echo) GD. The sequence parameters for the conventional images used in the study analysis were as follows:

**T1WI spin echo before and after (T1WIGD) contrast agent injection**: axial, FOV 220 × 165 mm, 24 slices, voxel size 0.43 × 0.43 × 5 mm (gap 1 mm), TE = 17.7 ms, TR = 2 524 ms, TI (inversion time) = 798 ms. The amount of contrast agent (gadodiamide 0.5 mmol/mL, Omniscan, GE Healthcare, US) was 0.2 mL/kg, with a maximum dose of 15 mL.

**T2WI spin echo PROPELLER**: axial, FOV 220 × 220 mm, 24 slices, voxel size 0.43 × 0.43 × 5 mm (gap 1 mm), TE = 95–97 ms, TR = 3 000 ms.

**3D-FSPGR GD**: axial, FOV 240 × 240 mm, 172 slices, voxel size 0.94 × 0.94 × 1 mm, TE = 3.2 ms, TR = 8.2 ms, TI = 450 ms.

The quantitative sequence is a multi-slice, multi-echo and multi-saturation delay qMRI technique for simultaneous measurement of R_1_, R_2_ and PD^26^, with the following parameters in this study:

**qMRI MAGiC**; axial, FOV 220 × 180 mm, 24 slices, voxel size 0.43 × 0.43 × 5 mm (gap 1 mm), ASSET acceleration factor 2. In total 8 images per slice were measured with TE = 22 ms or 95 ms, TR = 4 000 ms, TI = 170, 670, 1840 or 3840 ms. The scan time was 5:55 min, and the qMRI-series was obtained before and after contrast agent injection. The synthetic images created from the qMRI scan had the same settings as the corresponding conventional MR images.

After contrast agent injection spin echo images were acquired first followed by the 3D-FSPGR and lastly the qMRI.

### Post-processing and ROI placement

#### qMRI post-processing

The qMRI sequence yields quantitative maps of R_1_, R_2_ and PD, which are used for measurements and to create synthetic images matching the conventional images, using the same parameters as in the conventional images. The post-processing of the raw image dataset to create synthetic images was performed on a conventional computer using SyMRI 8 software (SyntheticMR AB, Linköping, Sweden).

#### ROI analysis

Conventional T2WI, T1WI, T1WI-GD, and 3D-FSPGR GD images and synthetic images with corresponding settings (synT1WI, synT2WI, synT1WI-GD) were transferred to the software MeVisLab version 2.7 (MeVis Medical Solutions AG, Bremen, Germany).

**Figure 1:**
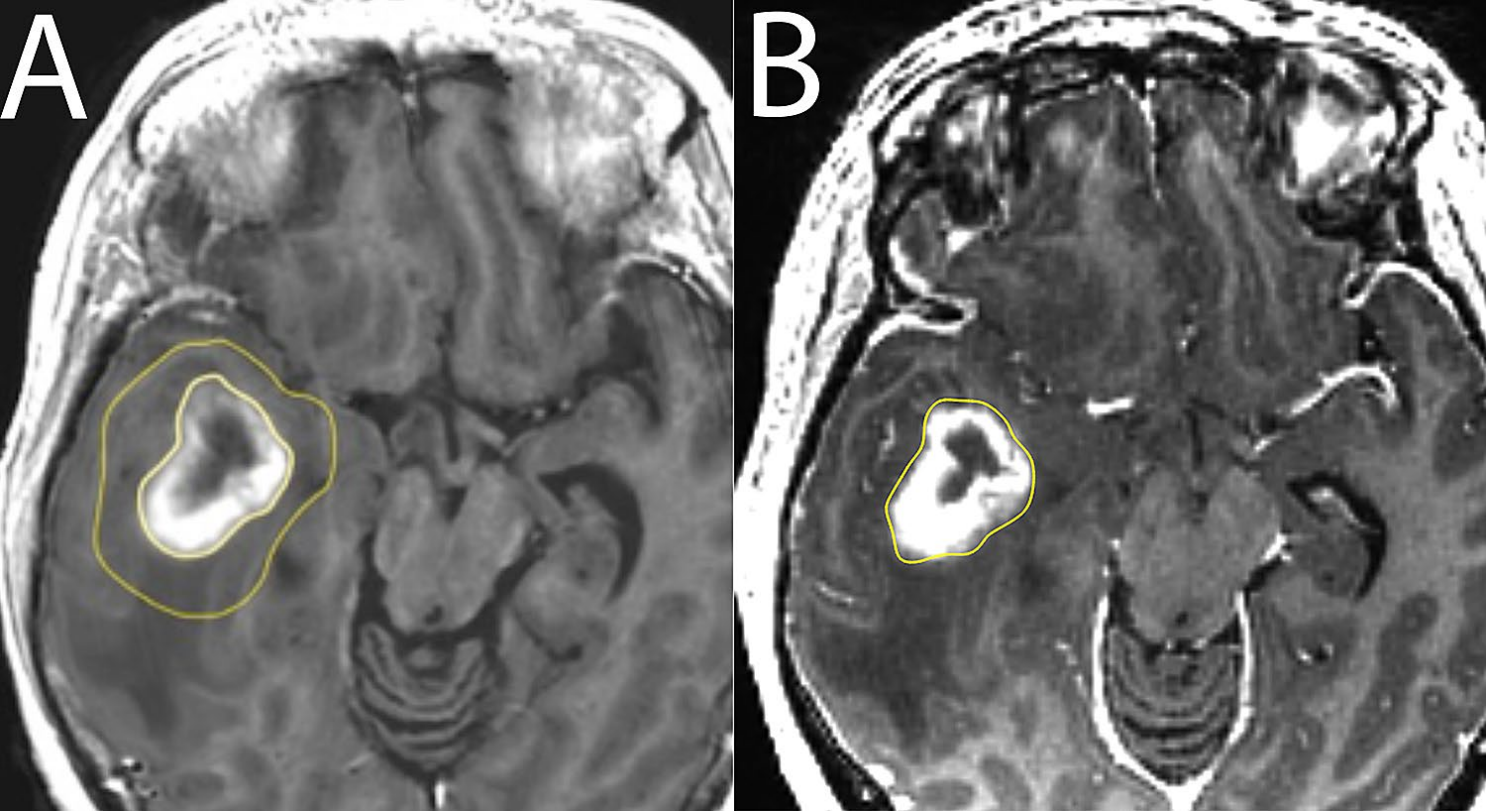
Tumour- and extended tumour-ROIs. An example of tumour delineating ROIs in synT1WI GD (**A**) and in 3D-FSPGR GD images (**B**), and an extended-tumour ROI in the synT1WI GD (**A**). The tumour ROI was subtracted from the extended-tumour ROI to analyse contrast enhancement in the peritumoral oedema in the synthetic images.

A neuroradiologist (IB) manually drew regions of interest (ROIs) for the analysis. The outer contrast-enhancing borders of the tumours were delineated (tumour-ROI) in the 3D-FSPGR GD images and in the synT1WI-GD (Fig. 1A) separately (Fig. 1B). The whole tumour was delineated slice by slice.

A second free hand extended ROI (extended tumour-ROI) was placed approximately 1 cm outside of the tumour-ROIs in the synT1WI-GD. Care was taken not to include contrast-enhancing structures, e.g. vessels or the choroid plexus (Fig. 1A).

The tumour volumes were calculated from the 3D-FSPGR GD images in MATLAB (MathWorks Inc, Natick, US). The conventional images were used for volume calculation due to their higher resolution.

Using the synthetic images, the peritumoral area was analysed by subtracting the tumour ROI from the extended tumour ROI to achieve a peritumoral ROI. In some tumours with a necrotic centre, an additional ROI was placed in the necrosis (necrosis ROI).

ROIs were also placed in synT1WI and synT1WI-GD in the normal appearing white matter in the corresponding lobe at the same level in the contralateral hemisphere (NAWM ROI). The NAWM ROI-size was approximately 1 cm in diameter. Similar sized ROIs were also placed in the distal peritumoral oedema at the periphery of the oedematous zone (distal oedema ROI). Hence there are three areas that were quantitatively compared: the peritumoral ROI, the distant oedema ROI, and the NAWM ROI. The tumour ROI and the necrosis ROI are presented in the descriptive histograms.

#### Calculation of R_1_-difference and PD

A transformation matrix was calculated for pre-GD to post-GD transformations by registration of synT2WI to synT2WI-GD, using the MeVisLab MERIT module with registration method set to “3D rigid” and similarity measurement to “SSD” (sum of squares of intensity distance). The transformation matrix was used to transform quantitative R_1_-maps from pre-GD space to post-GD space. Then, the quantitative difference in R_1_-relaxation due to contrast enhancement was calculated as the difference between the R_1_-maps post-GD and the transformed native R_1_-maps (R_1_-diff map). The R_1_-diff map is proportional to the GD concentration in the brain according to the relationship R_1_postGD − R_1_preGD = relaxivity × GD concentration^27^.

The ROIs were then applied to the R_1_-difference map and the PD map.

### Statistics

Descriptive histograms were calculated and graphed in MATLAB (MathWorks Inc, Natick, US). The mean and standard deviation were calculated for each ROI in each subject. A one-sided t-test was performed on the peritumoral ROI and the NAWM ROI; calculations were performed in Microsoft Excel.

All methods were carried out in accordance with relevant guidelines and regulations.

## Results

The volumes of the contrast-enhancing portions of the tumours, including necrotic centre when applicable, were calculated from the conventional 3D-FSPGR GD and are presented in Table 1.

The numbers of voxels in each ROI-type were as follows: tumour ROI median 26 530, minimum 4 250, maximum 120 658; peritumoral ROI median 58 804, minimum 15 292, maximum 96 082; distal oedema ROI median 319, minimum 0, maximum 899; NAWM ROI median 716, mininum 212, maximum 1 022.

**Figure 2:**
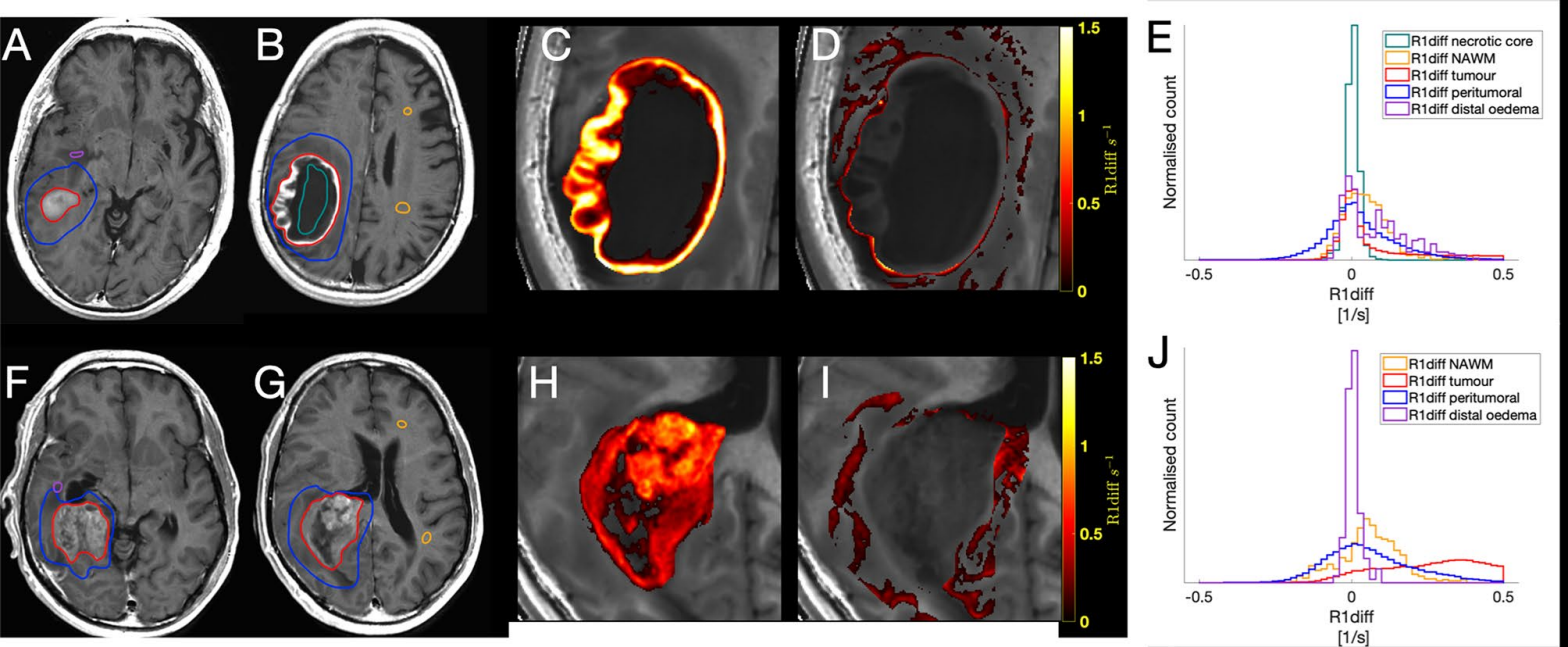
Glioblastomas and the contrast enhancement depicted as R_1_-difference. Two typical examples of glioblastomas. (**A**,**B**) synT1WI GD of a tumour with contrast enhancement in the periphery and a necrotic center. (**F**,**G**) synT1WI GD of a tumour with a more solid tumour appearance, exhibiting irregular contrast enhancement throughout the tumour. (**C**,**H**) zoomed images of the R_1_–difference in the tumour ROI. (**D**,**I**) show the R_1_–difference for the peritumoral ROI. (**E**,**J**) The corresponding histograms for R_1_-difference of each ROI; orange line for the NAWM, blue for the peritumoral ROI, red for the tumour ROI, green for the necrotic centre, and purple for the distal oedema. The R_1_-difference equals the contrast enhancement.

The tumours have a heterogeneous appearance on conventional MR-images, and also exhibit different relaxation patterns depending on the tumour structure. This is depicted in Fig. 2, which shows examples of two patients with glioblastomas with different appearances on MRI images as well as different relaxation patterns. Figure 2A,B (same patient) show a typical glioblastoma appearance with an intense contrast enhancement in the periphery, a more liquefied necrotic centre and peritumoral oedema. The contrast enhancement is depicted in the R_1_-difference graphs. For this tumour the R_1_-difference distribution has a maximum around zero corresponding to the necrotic centre (green line in 2E), and the contrast-enhancing part of the tumour contributes to the right-shifted, higher values on the x-axis (red line in 2E).

Figure 2F,G (same patient) show another appearance of a glioblastoma, with a more solid tumour portion, exhibiting irregular contrast enhancement throughout the tumour and a peritumoral oedema. The R_1_-difference graph for this tumour shows tumour relaxation values in a more dispersed, right-shifted distribution (red line in 2 J). Figure 2C,H show maps of the R_1_-difference in the tumour, which corresponds to the contrast enhancing part of the tumour. Figure 2D,I show the maps of the R_1_-difference in the peritumoral ROI.

**Figure 3:**
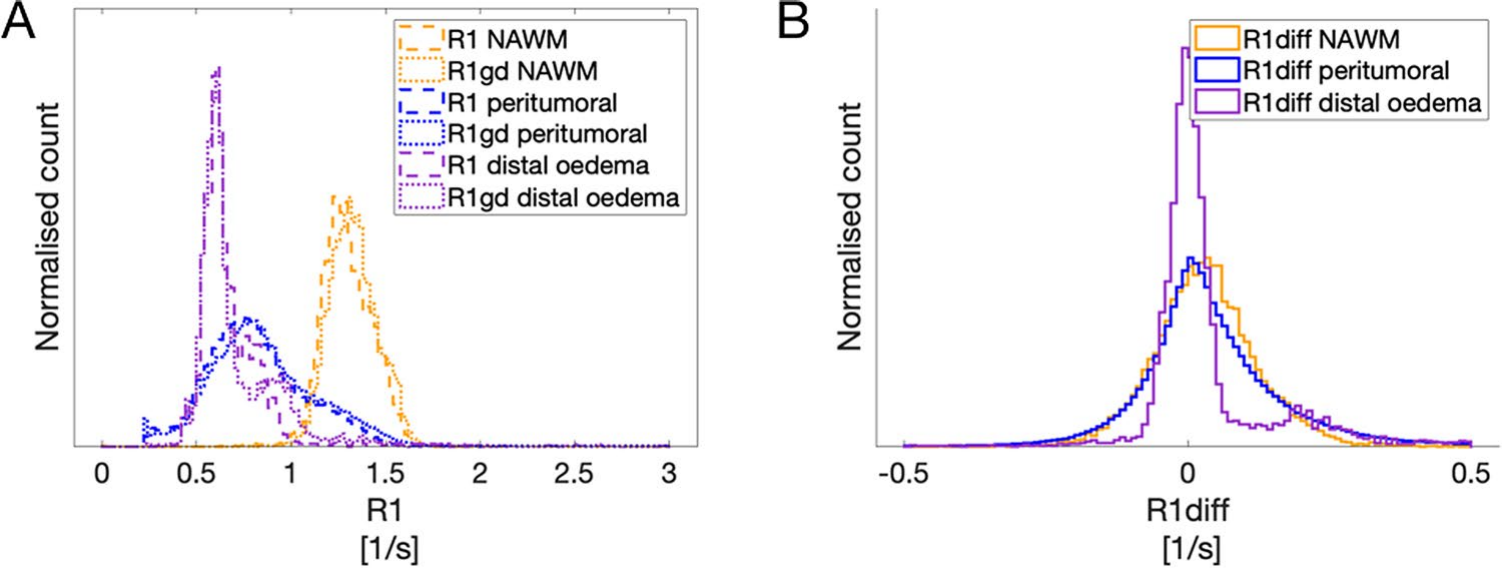
Graphs of R_1_ and R_1_-difference in tumour and peritumoral area. The R_1_ and R_1_-difference histograms of all voxels for all patients within respective ROI. (**A**) the values for R_1_ preGD (dashed line) and postGD injection (dotted line) are depicted for the NAWM, tumour and peritumoral ROIs. The lines shift to the right after contrast agent injection. (**B**) shows the R_1_-difference (contrast enhancement) histogram for the peritumoral (blue) and the NAWM (black) ROIs for all patients. The R1-difference is significantly higher in the peritumoral area compared to NAWM (p = 0.048).

In Fig. 3 the R_1_-difference is depicted for all voxels within respective ROI. Figure 3A shows the R_1_-values before (dashed line) and after (dotted line) GD-based contrast agent in the peritumoral area, in the NAWM, and in the distal oedema. R_1_-values are lower in the peritumoral area compared to the NAWM due to the higher water content of the peritumoral oedema. The R_1_-values are the lowest in the distal oedema for the same reason. The dotted lines are more right-shifted compared to the dashed lines due to the effect of gadolinium.

Figure 3B shows the contrast enhancement as the R_1_-difference for all tumours; in the peritumoral area (blue), in the NAWM (orange), and in the distal oedema (purple). The R_1_-difference overlaps, but the mean R_1_-difference is significantly higher in the peritumoral oedema compared to the NAWM (p = 0.048).

All R_1_-difference curves have values below zero, which would not be expected after injection of gadolinium based contrast agent. This is probably due to the distribution of the inherent noise in the data, but it could also partly be due to movement of the patient. However, movement of the patient would lead to both positive and negative R_1_-difference values and in mean the effect this will be cancelled out. Maps of the R1-differences, positive and negative, for the tumors and the peritumoral areas are provided in the supplementary figures.

**Figure 4:**
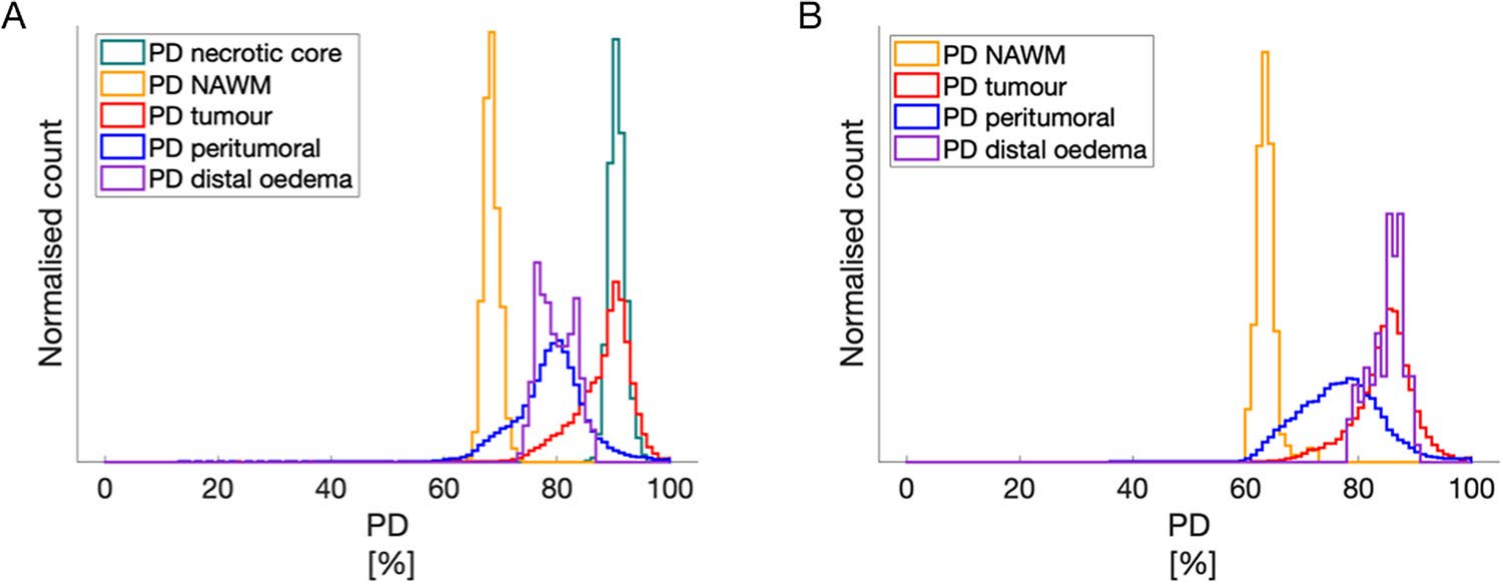
Graphs of PD for two different tumours. The PD histograms of the different ROIs in the patients in Fig. 2; 4A corresponding to 2A/B, and 4B corresponding to 2F/G.

Figure 4 shows the histogram for PD values of the different ROIs for the same patients as in Figs. 2, 4A corresponding to 2A/B and 4B corresponding to 2F/G.

**Figure 5:**
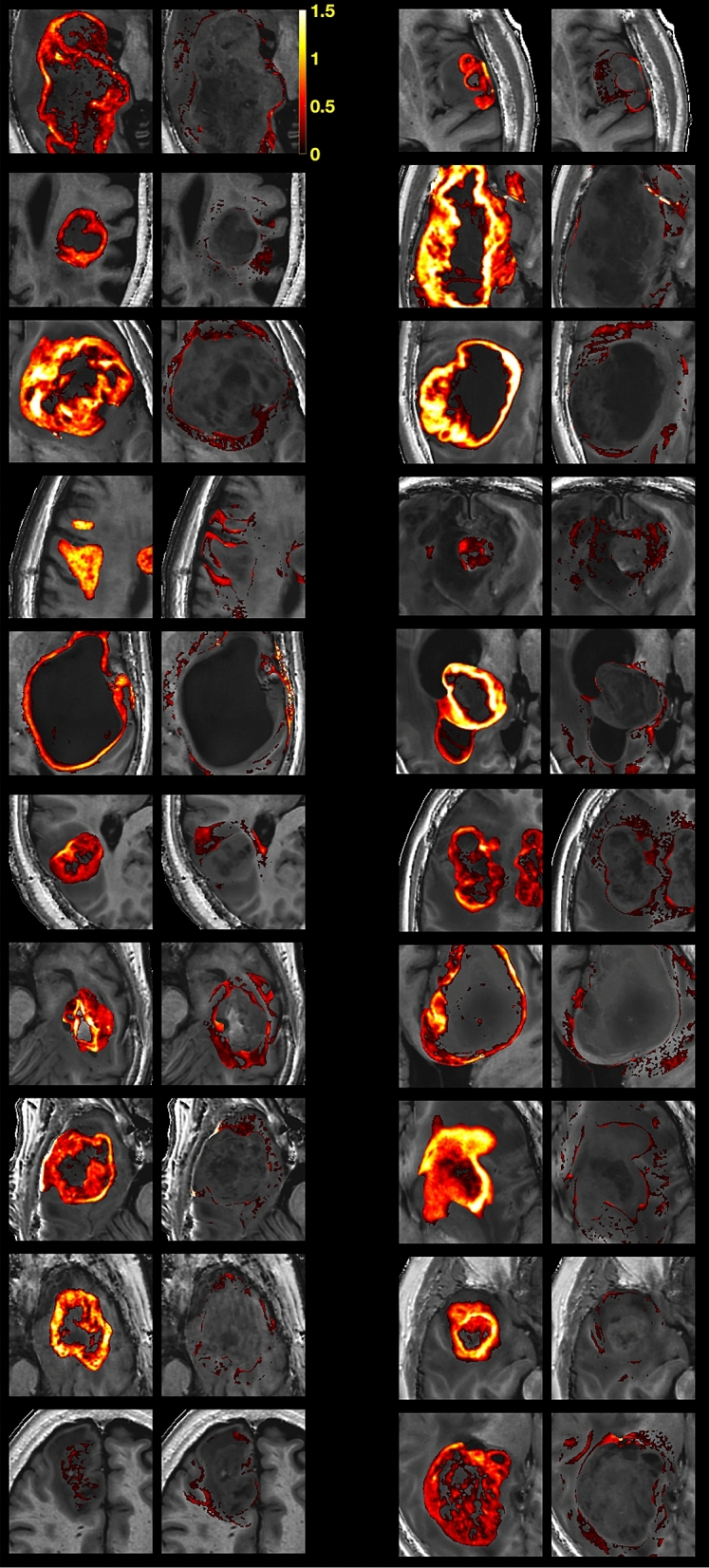
Maps of R_1_-difference. Paired R_1_-difference maps of the tumour (left) and the peritumoral ROI (right) of all patients. The threshold is set at values higher than NAWM + 1SD.

In Fig. 5, paired maps of the R_1_-difference of the tumour (left) and the peritumoral ROI (right) are shown for all patients. The threshold is set at values higher than group mean NAWM + one standard deviation.

Relaxation values before and after gadolinium based contrast agent injection in the peritumoral area, the distal oedema and the NAWM are shown in Table 2. There is a significant difference in R_1_ in all three tissues, indicating an effect of the contrast agent outside of the contrast enhancing part of the tumour.

**Table 2:** R_1_ before and after GD in peritumoral area, distal oedema, and NAWM.

	Pre-GD		Post-GD		
	Mean	SD	Mean	SD	P
Peritumoral	0.8	0.1	0.9	0.1	< 0.001
Distal oedema	0.69	0.17	0.74	0.22	0.019
NAWM	1.3	0.1	1.3	0.1	< 0.001

R_1_ values before and after GD-based contrast agent injection in the peritumoral area, the distal oedema, and in the NAWM.

Table 3 shows the mean and standard deviation (SD) of PD and of the R_1_-difference (R_1_ before gadolinium subtracted by R_1_ after gadolinium) in the ROIs of the peritumoral-ROIs compared to the NAWM-ROIs in synthetic images. There is a significant difference in the R1-difference between the peritumoral area compared to the NAWM (p = 0.048), with a higher R_1_-difference in the peritumoral area indicating a greater T1-shortening effect after gadolinium based contrast agent injection here, compared to NAWM. There is also a significant difference in PD value between the peritumoral area and the NAWM (p < 0.001), with a higher PD value in the peritumoral area as expected, due to higher water content in the oedema than in the NAWM.

**Table 3:** PD and R_1_-difference in peritumoral area compared to NAWM.

	Peritumoral		NAWM		P
	Mean	SD	Mean	SD	
PD	79.8	3.3	65.7	2.4	< 0.001
R_1_-diff	0.047	0.029	0.032	0.029	0.048

The R_1_-difference is a measurement of the effect of gadolinium on the tissue.

## Discussion

Visual assessment of the peritumoral area of malignant gliomas is a radiological challenge, since the diffuse, non-enhancing infiltration of these brain tumours cannot be visualized using conventional MRI^28^. The non-enhancing parts of the tumours are also a challenge during follow-up, since they have to be considered in the treatment evaluation^29^ but are difficult to detect. Clinically, this transitional zone between the contrast-enhancing part of the tumour and the peritumoral oedema is therefore of great interest, since the surgical treatment aims to resect as much as possible of the tumour without causing impairing sequelae to the patient. However, even with radical resection, tumour recurrence is in most cases localized to the resection margin^30^, and at present there is no conventional MRI technique implemented in standard practice to visualize the non-enhancing parts of the malignant gliomas. The MRI-technique used in this study is quantitative compared to conventional MRI, which relies on visual assessment. The benefits of a quantitative technique is that it is measurable and provides a basis for longitudinal comparison, since it is independent of scanner settings, inhomogeneity of the B1-field, and coil sensitivity profile.

In this study we report that it was possible to measure contrast enhancement in the peritumoral area, evident as a significant R_1_-difference after gadolinium, which could indicate that there is a subtle blood–brain barrier leakage present. This observation may be a result of diffuse tumour infiltration with a subsequently increased neo-angiogenesis, a process that also affects perfusion properties of the peritumoral tissue^31^.

These findings are in line with other studies. Quantitative MR sequences, such as T1- and T2-relaxometry, have been shown to have the capacity to detect non-visible tissue changes in the peritumoral oedema in malignant gliomas prior to surgery^24^. Moreover, such techniques have also been used to detect early tissue changes in glioblastoma recurrence during follow-up, before any changes were evident in the conventional images^32,33^. Additionally, the finding of non-visible peritumoral contrast enhancement is in line with the findings of Müller et al.^34^, using quantitative T1-mapping to detect peritumoral contrast enhancement invisible in standard MRI. They also showed that a decrease of this invisible contrast enhancement during treatment was prognostic for a favourable treatment response.

Ellingson et al. used conventional T1W GD subtraction maps with an automated method for detection of contrast enhancement. They examined patients during follow-up of glioblastomas and found an improvement in delineation and prediction of survival compared to conventional segmentation^35^. Conventional images, however, have the drawback of arbitrary intensity scaling, which makes it difficult to compare results between different scanners and different patients, a problem that can be solved by using a quantitative sequence, which actually measures the relaxation values of the tissue, like in this study.

Even in regions with an intact blood–brain-barrier there is some T1-shortening in the NAWM of the brain parenchyma following GD based contrast agent injection^36,37^. This physiological phenomenon is evident also in the values of NAWM and in the distal oedema in this study, which show an R_1_-difference after GD based contrast agent injection, but to a lesser extent than in the peritumoral area.

This study has some potential limitations. We hypothesize that our findings of an increase in peritumoral R_1_-difference could be due to infiltration of tumour into the peritumoral oedema. However, this study lacks the histopathological proof of correspondence with tumour infiltration.

When subtraction maps are used, the performance of the image registration procedure is important. The registration of quantitative R_1_ maps therefore benefits from the registration to synthetic T2WI, which to a large degree are not very affected by GD. Thus the registration of the quantitative values is, in our view, more robust compared to registration of the corresponding conventional T1WI. A limitation in this study is therefore that any subject motion will reduce the effective spatial resolution due to the influence of partial volume effect in the registration, especially in the z-direction (i.e. head-feet) because of the relatively thick slices that were used. Recently, a method for synthesizing a contrast-enhancement map based on post-contrast images only has been suggested^38^. This novel procedure would overcome the problem of possible patient motion and therefore make the technique even more robust.

Another challenge is that the tissue changes that we are interested in detecting are minimal and inhomogeneous, and it is therefore a major challenge to distinguish the pathology from the normal tissues, which also creates noise/variance during MR examination. This is perhaps most evident from the R_1_-difference graphs, in which the ROIs from necrosis and NAWM have slightly negative average difference-values, which is not intuitive, but is due to the inherent noise level.

The small number of patients is a limitation of this study, and the tumour classification is not made according to the latest WHO standard since it was not implemented at the time of patient recruitment. Since the inclusion criteria were based on visual assessment of a contrast-enhancing tumour with malignant appearance, the patient group is heterogeneous, ranging from the grade IV WHO tumours, to a grade II tumour. For future studies, it could be of interest to investigate how relaxation properties are correlated to new molecular markers, e.g. IDH-status. The analysis could also benefit from a quantitative sequence with isotropic voxels and thinner slices, for improved segmentation. This could aid in the continuing the work, to visualize these changes on an individual level.

In conclusion, the use of quantitative subtraction maps based on relaxometry makes it possible to detect contrast enhancement in the peritumoral oedema of malignant gliomas. We interpret this finding as a quantitative representation of tumour infiltration and the technique described here could therefore potentially be useful in the context of surgical planning. The procedure could then aid maximum resection of the tumour, while preserving areas affected only by peritumoral oedema, thereby potentially improving the outcome for the patient.

## Supplementary information

Supplementary Information 1.

Supplementary Information 2.

Supplementary Information 3.

## Data Availability

Datasets analysed in this study are not available due to patient confidentiality.
