# Supplementary material for: Quantitative MRI using relaxometry in malignant gliomas detects contrast enhancement in peritumoral oedema
Source: Sci Rep. 2020 Oct 22;10:17986. doi: 10.1038/s41598-020-75105-6 (PMC7581520; doi:10.1038/s41598-020-75105-6)
Supplement: Supplementary file 1 — Supplementary Information 1. [file 41598_2020_75105_MOESM1_ESM.docx]

**Quantitative MRI using relaxometry in malignant gliomas detects contrast enhancement in peritumoral oedema**

I Blystad, JBM Warntjes, Ö Smedby, P Lundberg, and E-M Larsson, A Tisell

Caption supplementary figures:

Supplementary figure 1:

In supplementary figure 1, zoomed images with maps of the positive R_1_-difference for the tumour as well as the peritumoral ROI are shown in the same image for all patients. The threshold is set at values above the group mean + 1 standard deviation of the NAWM ROIs.

Supplementary figure 2:

In supplementary figure 2 maps of positive and negative values of the R_1_-difference in the tumour (left) and the peritumoral ROI (right) are shown for all patients. Threshold for negative values is set at zero or lower, the threshold for positive values is set at values above the group mean + 1 standard deviation of the NAWM ROIs.
