# Supplementary figures and images for: Quantitative MRI using relaxometry in malignant gliomas detects contrast enhancement in peritumoral oedema
Source: Sci Rep. 2020 Oct 22;10:17986. doi: 10.1038/s41598-020-75105-6 (PMC7581520; doi:10.1038/s41598-020-75105-6)

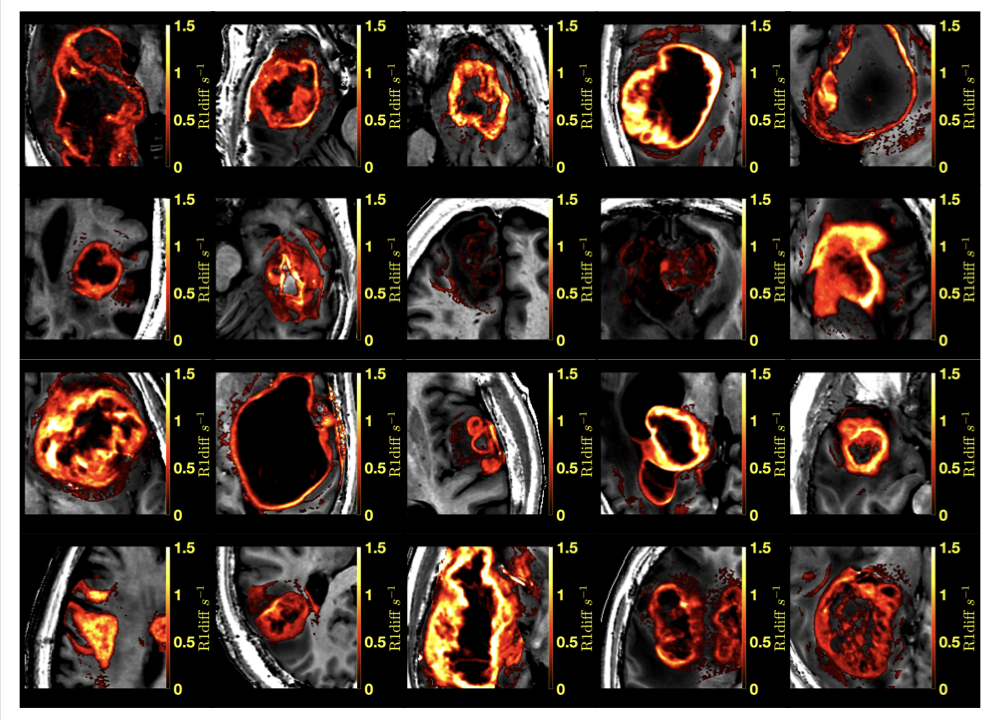

Supplement: Supplementary file 2 — Supplementary Information 2. [file 41598_2020_75105_MOESM2_ESM.png]

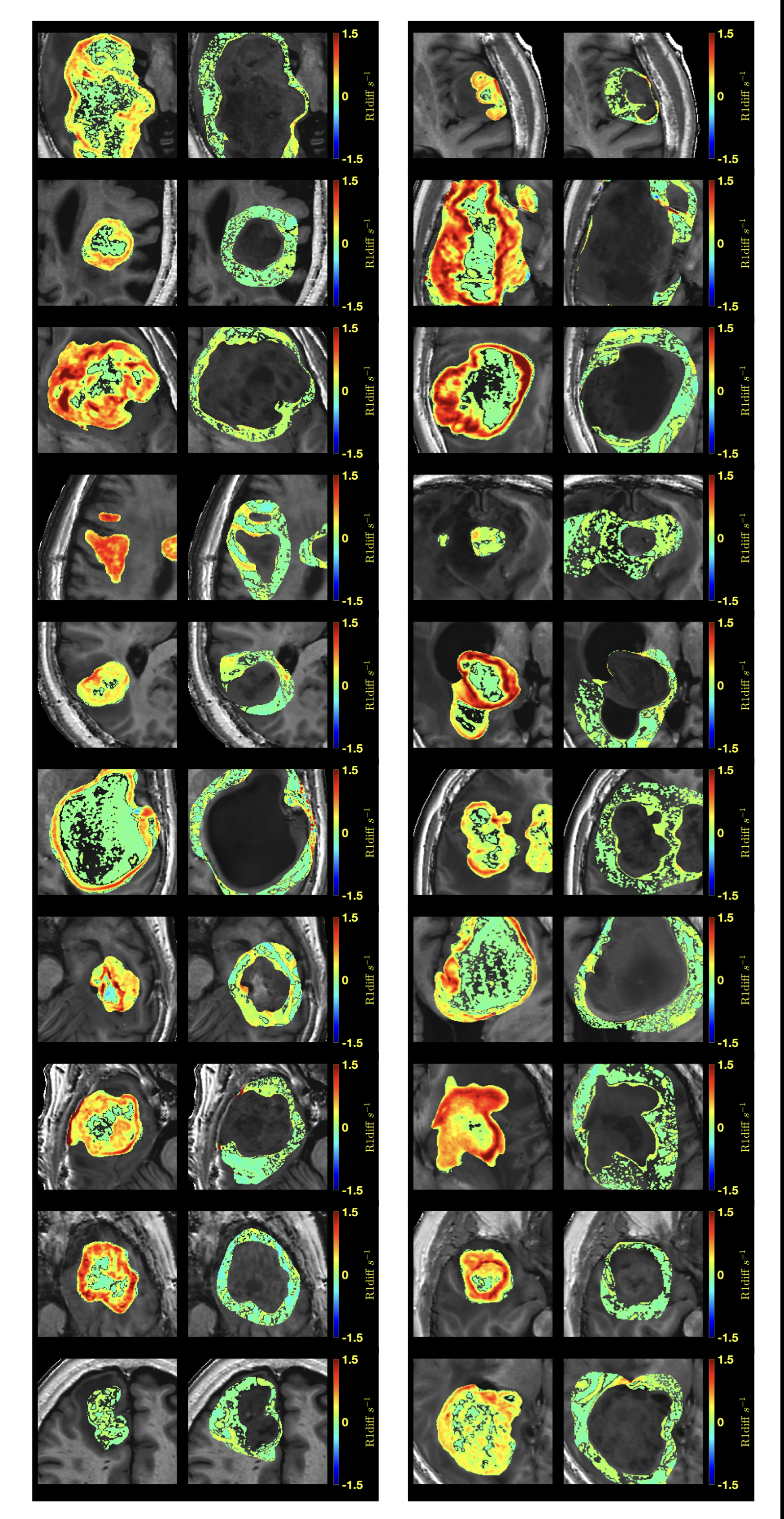

Supplement: Supplementary file 3 — Supplementary Information 3. [file 41598_2020_75105_MOESM3_ESM.png]
